# Design, fabrication and application of self-spiraling pattern-driven 4D-printed actuator

**DOI:** 10.1038/s41598-022-23425-0

**Published:** 2022-11-07

**Authors:** Siyuan Zeng, Yicong Gao, Hao Qiu, Junjun Xu, Jianrong Tan

**Affiliations:** 1 State Key Laboratory of Tribology and Institute of Manufacturing Engineering, Department of Mechanical Engineering, Tsinghua University, Beijing, China; 2 State Key Laboratory of Fluid Power and Mechatronic Systems, Zhejiang University, Hangzhou, China; 3 School of Mechanical Engineering and Automation, Zhejiang Sci-Tech University, Hangzhou, China; 4 Faculty of Engineering, The Hong Kong University of Science and Technology, Hong Kong, China

**Keywords:** Mechanical engineering, Engineering

## Abstract

Self-spiraling actuators are widely found in nature and have high research and actuator-application value in self-lock and self-assembly. Four-dimensional (4D) printing is a new generation additive manufacturing of smart materials and has shown great potential for the fabrication of multi-functional and customized structures. The microarchitecture design of a bilayer actuator could bring flexible and diversified self-spiraling behaviors and more possibilities for practical application by combing 4D printing. This work investigates the stimuli effects of fiber patterns and fabrication parameters on self-spiraling behaviors of the bilayer actuator via both experimental and theoretical methods. This work may potentially provide pattern design guidance for 4D-printed self-spiraling actuators to meet different application requirements.

## Introduction

Self-spiraling structures are widely distributed in nature, such as in snakes, octopuses, and vines, as such structures have the advantage of a small volume and large strain^1^. With the rise of the 4D printing concept^2^, the boundaries and connotations of traditional 3D printing have been expanded to enable the fabrication of a variety of complex structures^3^. Four-dimensional printing using anisotropic methods relies on structural and process design to facilitate manufacturing and offer more precise control. Thanks to years of research on smart materials and structure design^4–6^, there are now various methods to stimulate 3D shape changes of printed actuators in the temporal dimension^7^. In order to change the configured operation or function in response to an environmental stimulus^8^, one can activate the active material in a controlled manner, such as by using various materials to respond to different stimulation sources (light sources, heat sources, magnetic fields, electric fields, solution concentrations, etc.)^9–12^. Through the stimulation process of smart materials, 4D printing produce the expected deformation^13,14^. On the other hand, 4D printing also uses the design of printed actuators to change the stress–strain distribution during deformation and thereby control the deformation^15–17^. Moreover, actuators that utilize the anisotropic characteristics of the bilayer structure offer more possibilities to realize complex structures for 4D printing^18–20^. Multilayer 3D printing improves the flexibility of 4D-printed actuators, especially when using the fused deposition method and direct-ink-write, thus reducing the difficulty of fabrication^9,21–23^. As a result, self-spiraling structures can be fabricated independently of epitaxial actuators and allowed to deform autonomously with external excitation.

Previous studies found that the process parameters in various printing forms during printing have an effect on the shape-memory properties of the structures^24–26^. Under the 3D printing method of Fused deposition modeling (FDM), the filler filling density, printing temperature, printing speed, layer thickness, and printing width were shown to have a significant effect on the mechanical properties of printed structures^27–29^. For different specific structures of the same material, the trends and effects of printing parameters on mechanical properties are different^30,31^. When single bending is the main purpose of deformation, the incremental effect of printing speed is relatively small. The influence of printing temperature is greater when reciprocal memory behavior provides the main deformation^32,33^. The fiber pattern mainly affects the direction of deformation in all types of deformation. When shape memory polymers comprise the material, different fiber patterns can achieve different degrees of anisotropy in the same material space^34^. For 4D-printed self-spiraling structures, these issues are currently receiving little attention. Due to its basic mechanical properties, 4D printing based on SMP mainly changes the modulus of elasticity and the coefficient of linear thermal expansion^35^. Moreover, the trend of the influence of the printing process on the parameters of the bilayer actuator was previously verified^36^. This paper focuses on the effects of the printing process on the mechanical properties of self-spiraling structures, which include the basic properties and shape memory properties.

In addition to experimental studies, there is a need for developing simulation models to provide guidance in the design of mechanical properties. However, the simulation models used in current research often do not match actual deformation, often approximate models are built experimentally^37,38^. When new materials or structures are used, new experiments are required to correct the simulations. The microscopic simulation of printed patterns is also very difficult^39^. Studies need to further investigate the intrinsic relationship between printing parameters and mechanical properties if the existing simulation accuracy and operability are to be improved^40,41^. For the self-spiraling actuator, the printing angle is generally considered to be the most relevant quantity that controls the shape memory direction, but, in reality, the printing angle also affects the elastic modulus in a fully-filled case, which is related to the shape memory properties. Moreover, the printing temperature is high during operation of the SMP, which can affect the energy storage modulus.

Based on the above analysis, this work aims to systematically study the deformation mechanism and structural design of controlled self-spiraling actuators based on 4D printing. First, the deformation mechanism of a controllable self-spiraling actuator based on the thermodynamic intrinsic model is analyzed, and a simulation parameter model is proposed. Thermal excitation experiments were designed, and the law of change informing this deformation on the elastic modulus was obtained. Moreover, a shape memory performance index is introduced to explore the effects on different printing temperatures, to correct the variables in the thermodynamic model, and to provide guidance on the selection of printing parameters. A simulation of self-spiraling brakes with different printing angles under an optimal printing temperature was carried out by COMSOL and compared to the experimental fiber. Finally, based on the simulation model, a self-assembly process of the self-spiraling structure was realized using a self-spiraling actuator.

## Materials and methods

### Materials

The SMP material used in this study was Tough polylactic acid (PLA) manufactured by Ultimaker and stored under a vacuum to prevent degradation and reduce the effects of atmospheric humidity. The allowable parameters of the material in a printed state were measured by DSC (Differential scanning calorimetry, DSC) and DMA (Dynamic mechanical analysis, DMA) tests. The original parameter information was as follows: a glass transition temperature (T_g_) of 65 °C, a modulus of 3523 MPa at room temperature, a Tm of 183 °C, a tensile yield strength of 49.5 MPa, a tensile stress at fracture of 45.6 MPa, a flexural strength of 103 MPa, and a density of 1.24 g/cm^3^.

### Mechanical experiment

**Figure 1:**
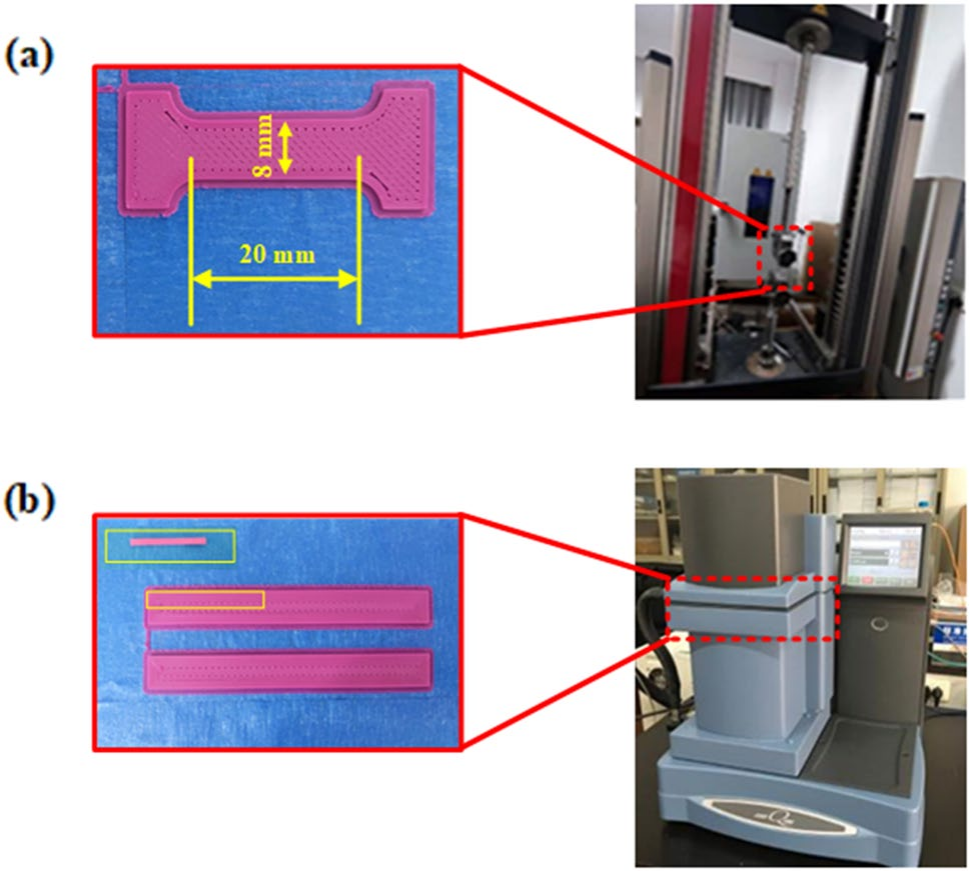
(**a**) DMA machine and printed sample; (**b**) tensile machine and printed sample.

DMA can test the glass transition temperature, storage modulus, loss modulus, loss factor of PLA materials with comprehensive functions and can also test changes in the PLA material properties within certain temperature and frequency ranges. Using the tensile mode, the test temperature for DMA ranged from room temperature to 130 °C. The temperature increase rate was 5 °C/min, the loading frequency was 1 Hz, the strain was 1%, and the preload force was set to 0.01 N. The printed sample strip was shown in Fig. 1a.

A tensile tester with an environmental chamber can test the tensile properties of materials under different temperatures and other conditions. The accuracy of the first level started from 4 N, and the accuracy of the 0.5 level started from 20 N; the size of the tensile specimen is shown in Fig. 1b. DMA Analyzer model number was Thermal Q800, and the model number of the tensile testing machine was Zwick Gmbh Z005.

### FEM simulations

In these simulations, printed PLA thin-layer 3D rectangles were placed into hot water to undergo thermally excited deformation. Thus, it was necessary to study the heat conduction of PLA-printed thin-layer 3D rectangles when heated in hot water. First, a single-layer three-dimensional rectangular actuator model was constructed with a length of 68 mm, a width of 8 mm, and a height of 1 mm. Since PLA materials did not exist in the case library, a material with general properties was established. This material’s thermal conductivity was set to 0.231 W/(m–K), its density to 1.24 g/cm^3^, its Poisson’s ratio to 0.3, and its specific heat capacity to 1985 J/kg*K.

The finite element simulation based on COMSOL uses the solid mechanics module, solid heat transfer module and transient solve, in modeling takes the solid mechanics module, solid heat transfer module and multiphysics field module for simulation, and sets the thermal expansion coefficient at the multiphysics field interface. In geometric modeling, the x and y plane is used as the reference plane to construct a three-dimensional two-layer rectangular thin-layer structure, the material direction of this structure, and the x-axis and y-axis angles of the global coordinate system, namely, in order to divide the reduced fine mesh to improve the computational time, and improve the computational accuracy, the length, width, and thickness of the thin-layer rectangular body are enlarged by 100 times. In the solid mechanics module, the hyperelastic material model is chosen to replace the original linear elastic material model, and rigid body motion suppression is set for the superstructure to control the degrees of freedom during the motion of the thin-layer rectangular body. In the solid heat transfer module, the initial values and heat fluxes of the thin-layer rectangular structure need to be defined. In the mesh division, the regular size is selected and the swept form is chosen, and the maximum mesh cell size is 0.68 m; the minimum mesh cell size is 0.122 m; the maximum cell growth rate is 1.5; the curvature resolution is set to 0.6; the narrow region resolution is 0.5; “distribution” is added below the swept “In the method settings of the transient solver, check “Include geometric nonlinearity” and set the time step to 0.2 s. The total time length is set according to the convergence time point.

### Self-spiraling behavior of the bilayer actuator

The structural parameters in the simulation include bilayer actuator width, length, and height, which were set by the 3D modeling software and fine-tuned in slicing software (CuraEngine 4.5 and Ultimaker Cura 5.1.0)^41,42^. The printing parameters are print fiber width, printing angle, and print layer height, which were adjusted in CuraEngine 4.5 and Ultimaker Cura 5.1.0. The print fiber width refers to the width of the trajectory applied by the print nozzle on the print platform, which is generally optional. To set this parameter, the range should be within 90–150% of the nozzle diameter. The printing angle refers to the angle between the trajectory applied by the print nozzle on the print platform and the direction of the long axis of the printed workpiece, with a value range of 0°–90°. The print layer height is the height of the trajectory applied by the print nozzle on the print platform, which is also the height of each print layer. The height is generally set to 20–80% of the print nozzle diameter size. The smaller the print layer height is, the higher the printing accuracy will be.

**Figure 2:**
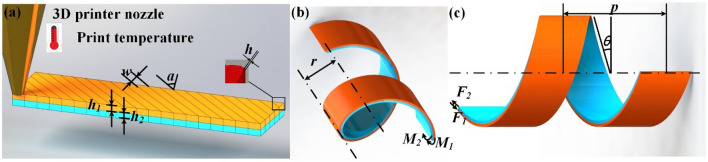
(**a**) Undeformed bilayer actuator; (**b**) oblique view of deformed bilayer actuator; and (**c**) front view of the deformed actuator.

The deformation of the bilayer actuator in 4D printing is shown in Fig. 2. The internal stress distribution in this process is shown in Fig. 2a. Here, the print height of the upper and lower layers is *h*_*1*_ and *h*_*2*_, respectively, and the print fiber width is *w*. The print height of each layer is *h*, and the printing angle is *a*. The deformed self-spiraling actuator is shown in Fig. 2b. The deformed bilayer actuator self-spiraling radius is *r*, and the bending moments of the upper and lower layers are *M*_*1*_ and *M*_*2*_, respectively. As shown in Fig. 2c, when the force (*F*_*1*_ and *F*_*2*_) direction have a certain angle, the bilayer actuator is deformed, after excitation, into a self-spiraling actuator, where *p* is the pitch, and *θ* is the gradient angle. The relationships in the self-spiraling actuator are shown as Eq. (1)^43^:

1 $$\left\{ {\begin{array}{*{20}c} {F_{1} = F_{2} = F} \\ {h_{1} = h_{2} = \frac{h}{2}} \\ {F_{1} h_{1} = F_{2} h_{2} = F\frac{h}{2} = M_{1} + M_{2} } \\ {F_{1} h_{1} = F_{2} h_{2} = F\frac{h}{2} = \frac{{E_{1} I_{1} + E_{2} I_{2} }}{\rho }} \\ \end{array} ,} \right.$$

where *F* is the size of the upper and lower force couples; $E_{1}$ and $E_{{2}}$ are the elastic modulus of the upper and lower layers, respectively; $I_{1}$ and $I_{{2}}$ are the rotational inertia of the upper and lower layers, respectively.

This equation obtains

2 $$r = \frac{2}{3}\frac{h}{{(\alpha_{2} - \alpha_{1} )(T - T_{0} )}},$$

where *α*_*1*_ and *α*_*2*_ are the coefficients of thermal expansion of the upper and lower layers, respectively; and *T* and *T*_*0*_ represent the heating temperature and initial temperature, respectively.

The printing parameters chosen in this paper are as follows: the print nozzle diameter was set to 0.4 mm, the print layer height was set to 0.08 mm, and the print fiber width was set to 0.4 mm. These parameters offer high manufacturability and good deformation effects^44^.

### Fiber pattern of the bilayer actuator

**Figure 3:**
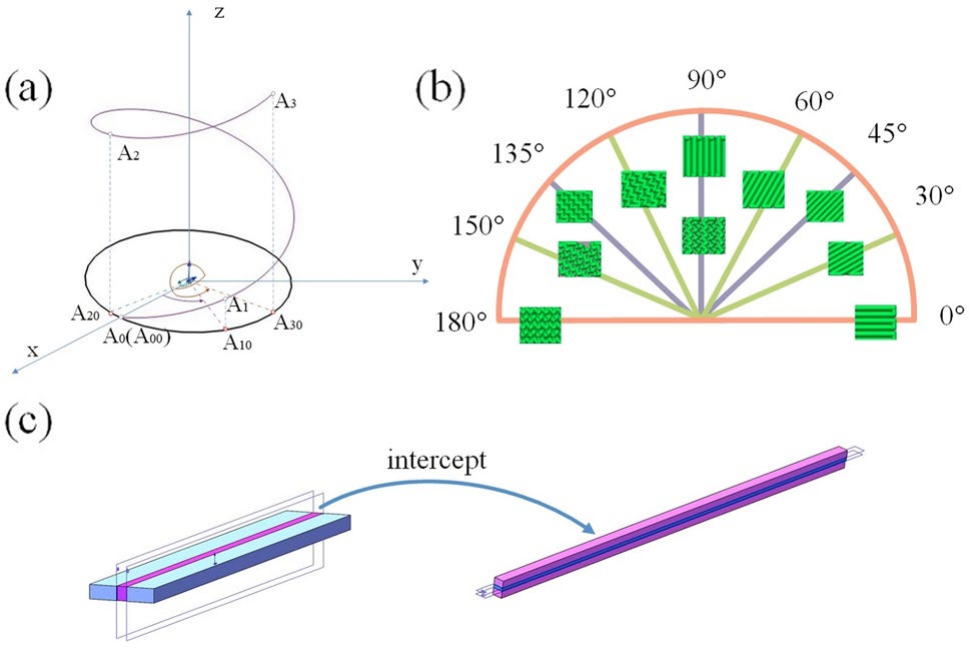
(**a**) Schematic diagram of the self-spiraling fiber; (**b**) different printing angles of FDM; and (**c**) the strain energy density function interception process.

As shown in Fig. 3a, the coordinate system *A*_*1*_* A*_*2*_* A*_*3*_ is a self-spiraling line, the top view of the part is a circle, and the radius of this circle is *r*_*0*_. Assuming that the starting polar coordinates coincide for the central axis of the self-spiraling and z-axis, the coordinates of point *A*_*10*_* A*_*20*_* A*_*30*_ on the line are (r_*0*_* θ z)*. When the point corresponds to the vertical coordinate, it has the relationship shown in Eq. (3).

3 $$\left\{ \begin{gathered} \begin{array}{*{20}c} {x = r\cos \theta } \\ {y = r\sin \theta } \\ {z = \theta \cdot b} \\ \end{array} \hfill \\ r = \frac{{r_{0}^{2} + (p/2\pi )^{2} }}{{r_{0} }} \hfill \\ \end{gathered} \right.,$$

The two images at 90° correspond to two linear print patterns, wiggle and linear pattern. Since the print pattern in the interval 0–180°is in fact specific only for 0°–90°, 90°–180°contains repeated stress phenomena, so two deformation trends are given at 90°. 90°–180° is illustrated as wiggle.

For the bilayer actuator in FDM, different fiber patterns were chosen to generate moments and force couples using the thermal expansion between the fiber patterns. In order to investigate the direction of the force coupling generated by the printing angle and the physical characteristics of the actuator, we selected a straight fiber and wiggle fiber with a 100% filling rate and 0°–180° for each angle. Ss shown in Fig. 3b, 0°–90° for straight fiber, 90°–180° for wiggle fiber. As shown in Fig. 3c, due to the printing angle, a small long strip is intercepted in the actuator width direction such that the strip’s length is equal to the width of the thin-layered 3D rectangular body. The intercepted long strip is then intercepted perpendicular to the z-axis, and the potential energy of each small surface piece is integrated over the z-axis. The energy density function can be expressed using Eq. (4):

4 $$\prod { = }f^{ + } :\left. \gamma \right|_{{z = \frac{{h_{2} }}{2}}} + f^{ - } :\left. \gamma \right|_{{z = - \frac{{h_{1} }}{2}}} + \int_{{z = - \frac{{h_{1} }}{2}}}^{{z = \frac{{h_{2} }}{2}}} {\frac{1}{2}\gamma :D:\gamma dz} ,$$

where *f*^+^ and *f*^-^ are the surface stresses at the middle of actuator; *D* is the fourth-order stiffness tensor; and *γ* refers to the elastic deformation tensor, the anisotropic bending curvature strain, and the uniform strain along the z-axis direction.

The deformation model of the self-spiraling actuator can be constructed by analyzing Eqs. (2), (3) and (4). Here, the deformation direction is basically perpendicular to the printing plane, and the angle is consistent with *α*. However, the relationship between the yield strength and elastic modulus is missing. Based on the force analysis, the deformation direction is basically perpendicular to the printing plane with an upward angle, but due to the mechanical properties imposed by 3D printing, the material’s deformation needs to be further determined to explore the relationship between its elastic modulus and the print angle.

In order to describe the relationship between the printing angle and these parameters, the upper layer of the bilayer actuator used in this paper was set as a wiggle fiber of 0°, while the lower layer was 0°, 30°, 45°, 60°, 90°, 120° and 135°, respectively. The force direction of the straight fiber coincided with the direction of the fiber print, with a slight wiggle of 10°–15°. At the same time, in order to obtain deformation process data, tensile experiments at 35 °C, 45 °C, and 55 °C were conducted, and the printing temperature was fixed at 195 °C.

### Fabrication parameters of the bilayer actuator

Fused deposition modeling (FDM) is a 3D additive process with a large range of applicable materials and a simple working principle, but this technique’s surface-forming accuracy is not high. Moreover, this technique creates a printing texture and is not suitable for printing large workpieces. This technology uses roller friction to roll the filamentary thermoplastic material into the heating mechanism, which is then heated by an electric heating plate and can reach a temperature of 220 °C. The melting point of thermoplastic materials is generally lower than 220 °C, so the filamentary material melts in the heating mechanism and is extruded by the print nozzle, with the extrusion pressure delivered by the roller mechanism. This repeated movement finally completes the target actuator molding. The 3D printer used in this paper was an Ultimaker2 Extend + .

In FDM printing, the PLA wire heated by the print nozzle is applied to the print platform and immediately cooled to the print platform temperature, a process in which the internal stress and stored strain energy are frozen in the frozen phase. During the deformation recovery process, the stored strain energy is released in the form of internal stress, which drives shape memory deformation and allows the SMP to recover from deformation.

The PLA bilayer actuator based on FDM, which can be called anisotropic, shrinks along the direction of the printing angle of the upper actuator and expands in the direction of the vertical printing angle when heated^35^. Anisotropic materials have properties that change with their orientation. The material properties of isotropic materials can be defined with the elastic modulus and Poisson's ratio using a relationship such as that in Eq. (5):

5 $$\{ \sigma \} \, = \,\left[ D \right]\; \cdot \{ \varepsilon \} ,$$

where {*σ*} is the stress vector, which characterizes the stress on the material in all directions, [*D*] is the 6th order elasticity coefficient matrix, and {*ε*}is the strain tensor.

Orthotropic anisotropic materials have three symmetrical surfaces with the elastic matrix outlined in Eq. (6), where the unknown variables can be obtained from the storage modulus and Poisson’s ratio. The above parameters can be obtained from a DMA test, and the relationship between the storage modulus and the printing temperature will be discussed in this paper.

6 $$\left[ D \right]{ = }\left[ {\begin{array}{*{20}c} {D_{11} } & {D_{21} } & {D_{31} } & {} & {} & {} \\ {D_{21} } & {D_{22} } & {D_{32} } & {} & {} & {} \\ {D_{31} } & {D_{32} } & {D_{33} } & {} & {} & {} \\ {} & {} & {} & {D_{44} } & {} & {} \\ {} & {} & {} & {} & {D_{55} } & {} \\ {} & {} & {} & {} & {} & {D_{66} } \\ \end{array} } \right].$$

In order to investigate the relationship between different printing temperatures and storage moduli, according to the printing temperature range of PLA, we selected temperatures of 195, 200, 205, and 210 °C and a printing angle fixed at a 0° recline. All the samples were tested at least two times, and the experiment’s results were repeatable.

## Results and discussion

### The effect of printing angle on the experiment

**Figure 4:**
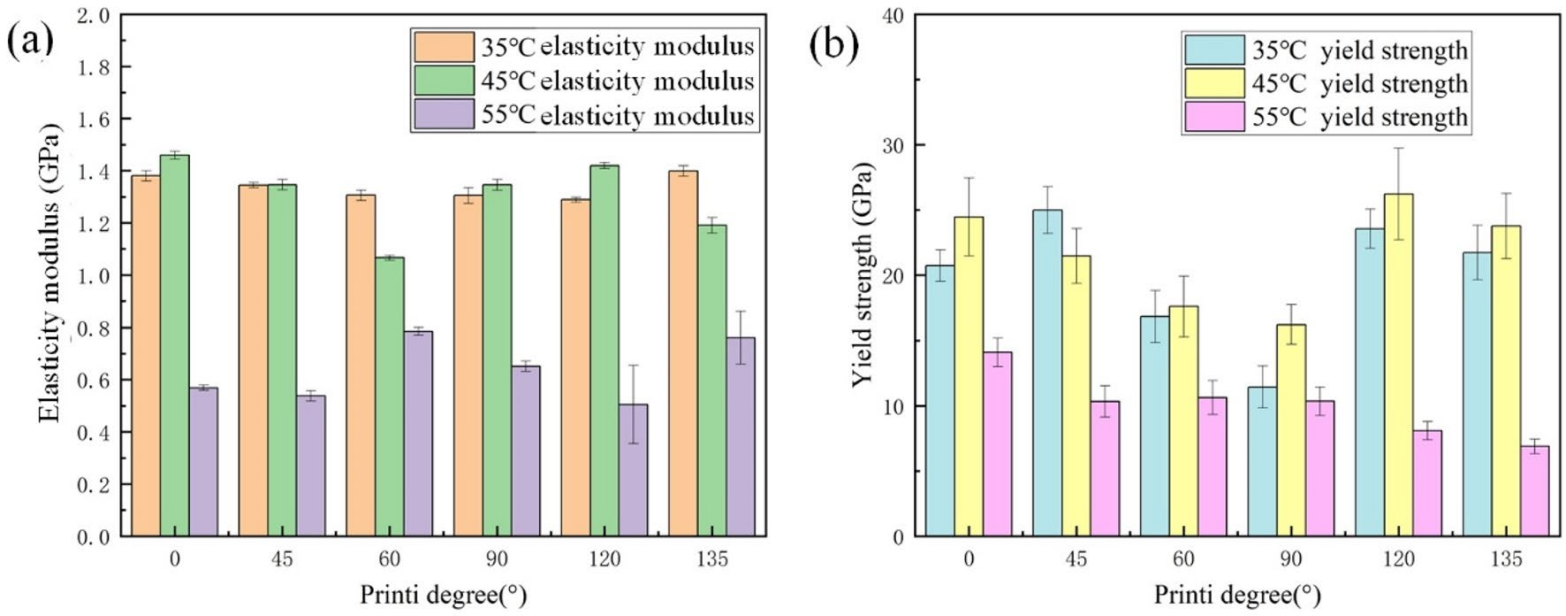
(**a**) Elasticity modulus for each temperature workpiece; and (**b**) yield strength of the workpiece at each temperature.

Figure 4 shows the measurements of the elasticity modulus and yield strength in the constant temperature tensile process. Figure 4a shows the stored energy modulus, and Fig. 4b shows the yield strength at 35, 45 and 55 °C. At 35 and 45 °C, the PLA print samples with 120° and 135° printing angles presented higher yield strength than 60° and 45°, and at 55 °C, the PLA print samples with 60° and 45° printing angles presented greater yield strength than 120° and 135°. This also verified that the synthetic stress angle of the wiggle pattern did not extend along the short or long axis but instead had a certain angle with the long axis. The yield strength of the PLA printed strips decreased gradually with temperature, but the yield strength at 35 °C was smaller than that 45 °C in the test. As the ambient temperature increased, the elastic modulus of the PLA printed strips gradually decreased from an average elastic modulus of 1.3373 GPa at 35 °C, to an average elastic modulus of 0.635 GPa at 55 °C, and then to an average elastic modulus of 0.004 GPa at 70 °C. At 70 °C, the tensile modulus and yield strength reached its minimum. The elastic modulus and yield strength obtained from the test using a print sample with a 0° printing angle were 0.004062762 GPa and 0.474794331 MPa, respectively, when the material was near the glass transition temperature.

When the printing temperature is certain, the larger the angle is between the printing angles, the greater material’s tensile elastic modulus and yield strength. When the stretching temperature reached the street glass transition temperature, the change in stretching presented a non-linear trend due to the transformation of the internal state of the material. In the measurable range of room temperature, while the workpiece was still in a glassy state, its elastic modulus and yield strength were inversely proportional to the size of the clamping angle, where the smaller the angle, the larger the modulus. When the glass transition temperature was approached, the modulus of elasticity and yield strength became proportional to the size of the angle, where the larger the angle, the larger the modulus. The material modulus obtained in this subsection provided the basis for the finite element simulation.

### Determination of the response rate of the self-spiraling actuator

**Figure 5:**
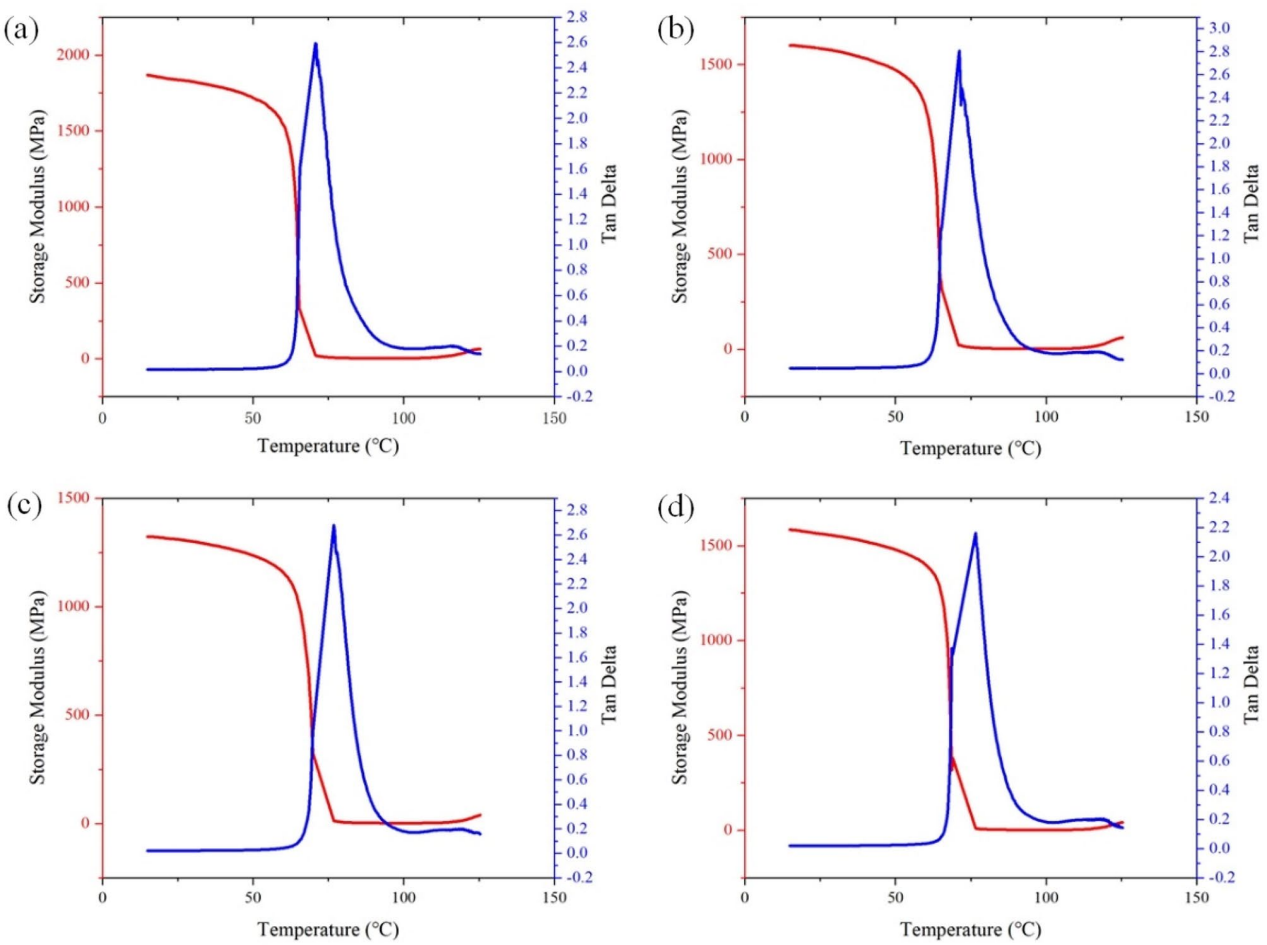
(**a**) 195 °C printing temperature energy storage and Tan Delta; (**b**)200 °C energy storage and Tan Delta; (**c**) 205 °C printing temperature energy storage and Tan Delta; and (**d**) 210 °C printing temperature energy storage and Tan Delta.

Figure 5 shows the experimental DMA curves of the workpiece at print temperatures of 195, 200, 205, and 210 °C. The figure contains data on the energy storage modulus and Tan Delta, where Tan Delta is the ratio of the energy storage modulus to the loss modulus, and the peak occurs when the T_g_ is reached. The T_g_ values of the four experimental groups were 70.7, 71.63, 76.72, and 76.82 °C. As the printing temperatures increased, the glass transition temperatures also increased—all above the initial glass transition temperature of 65 degrees for PLA. When the printing temperature exceeded 200 degrees, the magnitude of the change in the physical properties diminished as it approaches its melting temperature.

The storage modulus curve is divided into a high temperature zone (75–130 °C) and a low temperature zone (15–60 °C). In the low temperature zone between 15 and 60 °C, the material was glassy (similar to glass under the action of external forces producing small deformation), and the storage modulus became larger. From 60 to 75 °C, the material rapidly changed from a glassy state into a high elastic state—that is, during the glassy body transformation process, the storage modulus decreased rapidly. In the high temperature zone from 75 to 110 °C, the material was in a high elastic state (similar to rubber, the deformation produced under the action of external forces was larger); in the late part of this temperature zone, the deformation remained unchanged, and the storage modulus was low; when the temperature continued to rise above 110 °C, the material assumed a viscous flow state. The deformation at this stage was not reversible, and the storage modulus increased again, but the trend was slower.

By collating the maximum value of the storage modulus in the low temperature region with the maximum value of the storage modulus in the high temperature region, the glass transition temperature, and their corresponding loss factors at different printing temperatures, we obtained the changes in the parameters under different printing temperatures. The maximum value of the storage modulus in the low-temperature region decreased with an increase in printing temperature, reaching a minimum value of 1324 MPa at 205 °C and slightly increasing at 210 °C. The maximum value of the storage modulus in the high-temperature region presented a similar trend, reaching a minimum value of 38.85 MPa at 205 °C and slightly increasing at 210 °C. The maximum value of the storage modulus in the high-temperature region decreased with an increase in the printing temperature, reaching a minimum value of 1324 MPa at 205 °C and slightly increasing at 210 °C. The energy storage modulus also increased slightly at 210 °C due to this temperature’s close proximity to the material’s melting temperature, which also caused the change in the trend of T_g_.

In order to explore the shape memory properties of the material at different printing temperatures, we introduced the fixation rate *R*_*f*_, the expected recovery rate *R*_*r*_, and the comprehensive performance indicator *R*, which have the following relationships^45^:

7 $$\left\{ \begin{gathered} R_{r} = \varepsilon_{1} /\varepsilon_{0} \hfill \\ R_{f} = \left( {\varepsilon_{0} - \varepsilon_{2} } \right)/\varepsilon_{0} \hfill \\ R \, = R_{r} \times R_{f} \hfill \\ \end{gathered} \right.,$$

where *ε*_1_ is the strain at the glass transition temperature, *ε*_2_ is the strain under the DMA pre-processing average strain, and *ε*_3_ is the average strain in the subsequent process of DMA.

**Figure 6:**
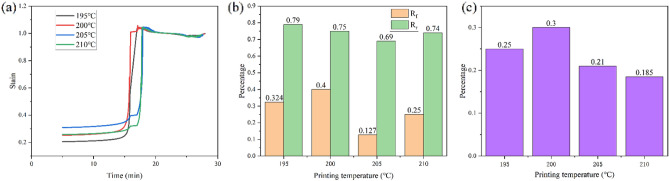
(**a**) DMA strain at different printing temperatures; (**b**) the R_r_ and R_f_ at different printing temperatures; and (**c**) the R at different printing temperatures.

**Figure 7:**
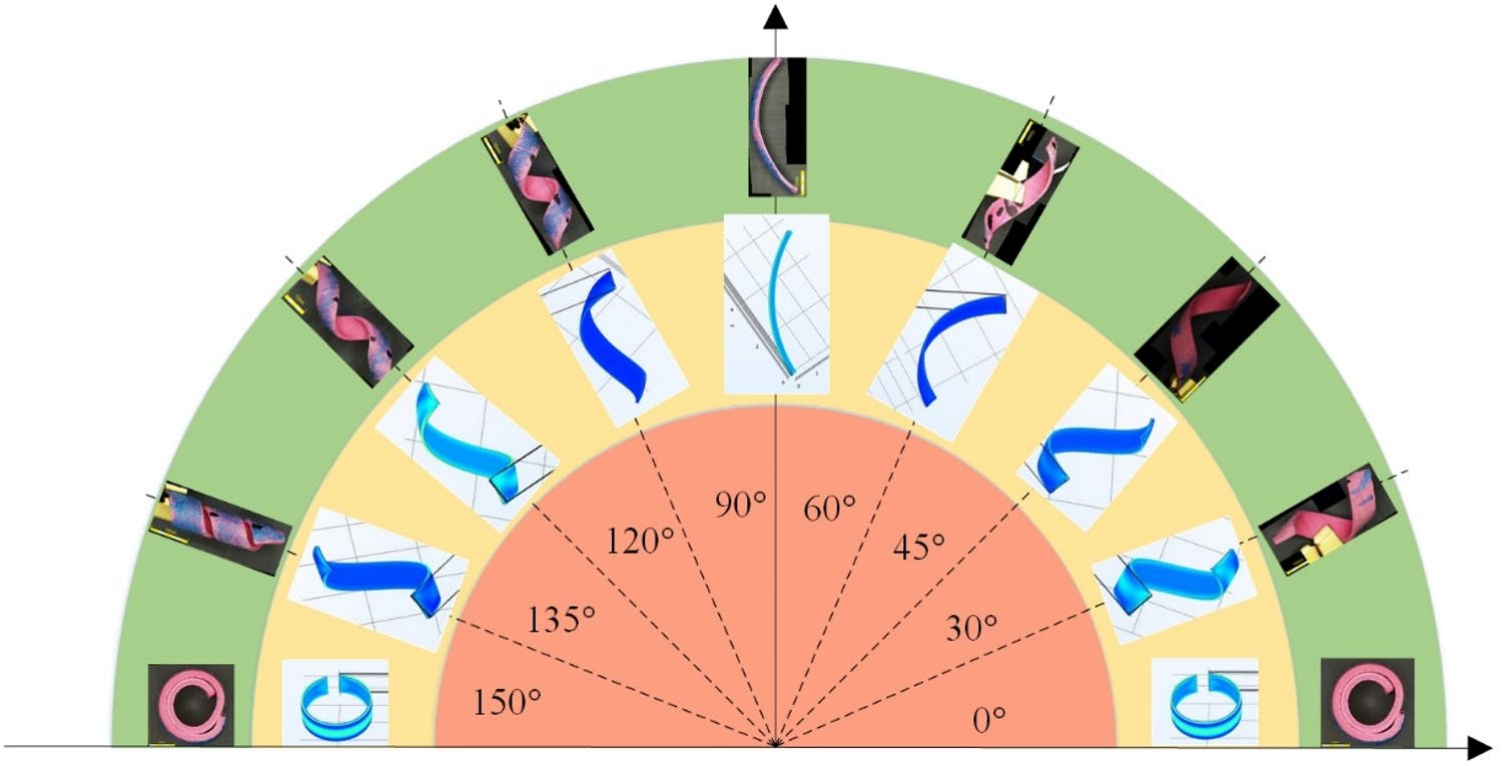
Simulation and experiment for different printing angles.

Figure 6 shows the strain process in the DMA experiment along with the arithmetic results from Eq. (7). As shown in Fig. 7b, the Rr decreases as the printing temperature rises. Here, the temperature is shown to rise at 210 °C in fiber with the trend in the glass transition temperature. Rf reaches a maximum value of 0.4 at 200 °C and then decreases to 0.127 and rises again to 0.25 at 210 °C. According to the comprehensive performance, the R value reaches a maximum value of 0.3 at 200 °C, 200 °C has the largest R-value in the experiment, so it has the best shape memory effect, indicating that the best shape memory performance was obtained at a printing temperature of 200 °C. The print temperature was shown to have a significant effect on the shape memory behavior, but the trend of each indicator near the melting temperature was anomalous and did not match the overall trend. A better shape memory effect can facilitate 4D printing with better deformation effects.

### Numerical simulation of the deformation of a self-spiraling actuator

Based on the analysis results in Sect. “The effect of printing angle on the experiment” and “Determination of the response rate of the self-spiraling actuator”, 200 °C was chosen as the printing temperature, and the conditions of the upper layer in Sect. “Mechanical experiment” and “Fiber pattern of the bilayer actuator”were adopted as the workpiece manufacturing parameters. The lower layer selection at 0° was adopted as the wiggle pattern. The size of the rectangular bilayer structure was 8 × 68 × 1 mm. Actuators were stimulated in a water bather and the heating temperature of the water was 85.5 °C. The simulated self-spiraling actuator corresponding to different angles and the experimentally obtained self-spiraling actuator are shown in Fig. 7. The angle of the inner red circle in the figure represents the printing angle and the angle relative to the lower layer. The yellow area in the middle corresponds to the simulated self-spiraling deformation shape. The green area of the outer circle corresponds to the self-spiraling actuator obtained by printing the upper layer angle. The simulated self-spiraling actuators were observed and imaged by optical digital microscope (Olympus DSX 110 with 3.5objective lens).

As shown Fig. 7, when the printing angle is 0°–90°, the self-spiraling structure obtained from the simulation is rotated left in the vertical printing plane, and the pitch increases with an increase in the angle; when with the printing angle is 90°–180°, the self-spiraling structure obtained from the simulation is preferred in the vertical printing plane, and the pitch decreases with an increase in the angle. Here, when the angle with printing is 0° and 180°, the pitch of the self-spiraling structure obtained by simulation is 0°, and the final deformation of the bilayer actuator is 90° when the final deformation of the bilayer actuator forms a ring. When corresponding to the printing angle, the pitch of the self-spiraling structure obtained by simulation is infinity, and the final deformation of the bilayer actuator produces bending deformation. In the simulated deformation, the trend of the structural parameters is the same as that of the self-spiraling structure parameters obtained from the test.

The stress in the middle region was observed to be greater than that at the edge during the simulation. This difference is due to the fact that the thermal expansion coefficient of the bilayer structure is equivalent to that of the lower layer; thus, the thermal strain of the lower layer is significantly greater than that of the upper layer when heated, and the strain of the bilayer structure as a whole must be equal at the joint surface. This bilayer structure generates tangential stress at the joint, thus making the stress in the middle region greater than that in the edge region.

### Application

**Figure 8:**
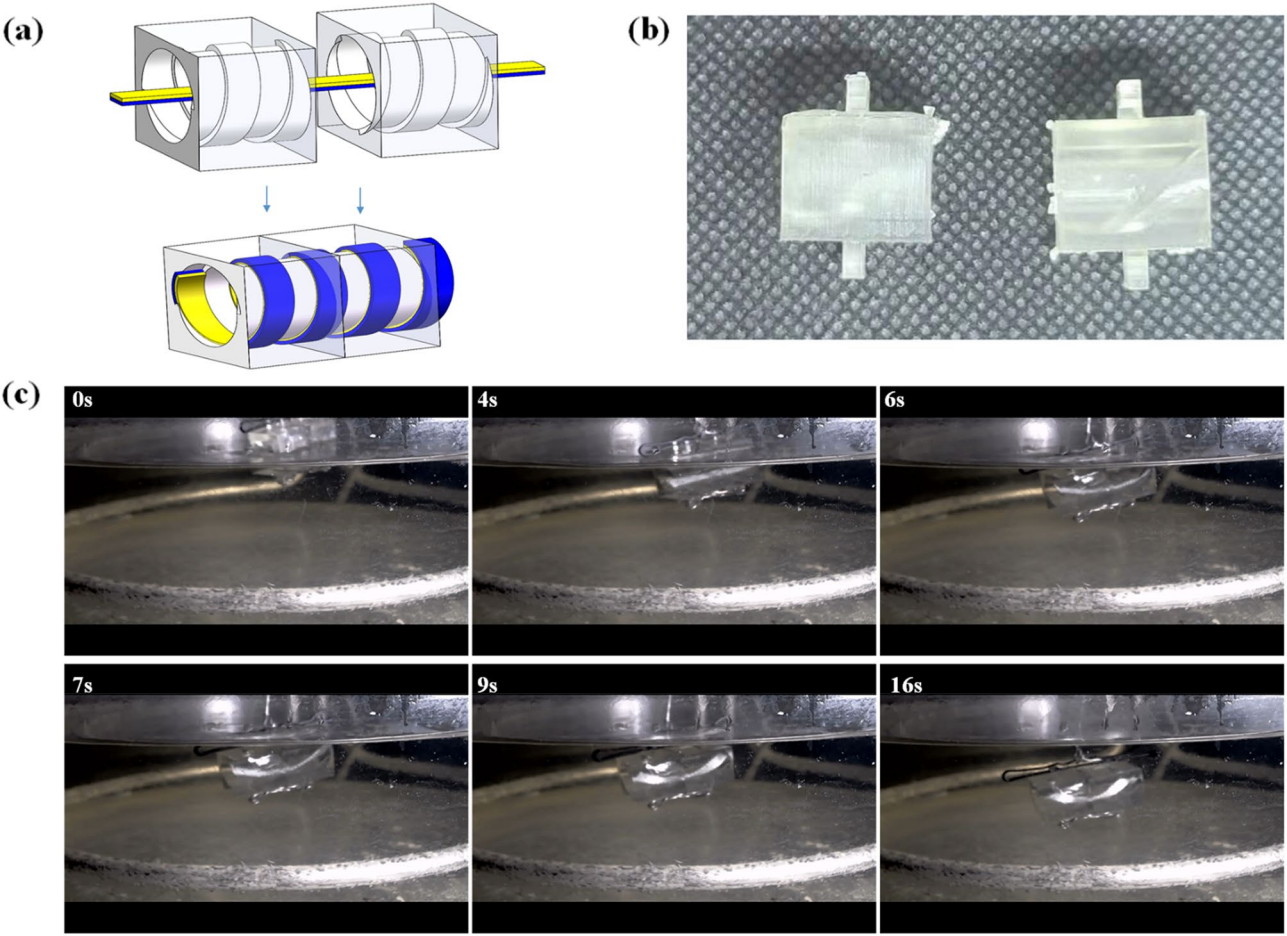
(**a**) Self-assembly schematic; (**b**)two bonding structures; and (**c**) articulation process.

For the assembly test, we selected a material size of 4 × 40 × 1.5 mm and a printing temperature of 200 °C. The upper layer was 30 °C with a straight grain, while the lower layer was 0 °C with a wiggle grain. Using the elastic material PLA, the two structures were docked with a self-spiraling brake by reserving the notches inside. The radius of curvature under this condition is 4.11 mm and the pitch is 32 mm. In order to make the self-assembly successful, the thread is selected with 30 mm pitch and 8 mm diameter, the stimulation temperature is 90℃. The experimental procedure is shown in Fig. 8.

The preceding section showed that a printing temperature of 200 °C provides better shape and performance, while a printing angle of 30 °C provides a better pitch-to-self-spiraling radius ratio. The sample was deformed and the deformation result was as expected, and the pitch was successfully embedded. Using the water-bath heating method, the two self-spiraling brakes were first placed in the middle of the two structures. After heating, the two structures were observed to join together by internal threads. Due to the buoyancy of the water bath heating and the uneven effect of heat transfer, the gap is slightly larger compared to the simulation.

## Conclusion

This paper explored the mechanical behavior of 4D-printed self-spiraling structures. An FDM-based printing method-focusing on the shape memory polymer bilayer actuator-was modeled, experimentally analyzed, and simulated, with special focus placed on the printing angle and printing temperature. The results showed that the printing angle not only determines the deformation trend in 4D printing but also affects the elastic modulus. The extrusion process of the self-jet nozzle is the process that imposes shape memory behavior. In this study, a 200 °C printing temperature offered the best shape memory performance. We also simulated different printing angles, and the trends were found to be the same as the actual test results. Finally, we conducted a self-assembly experiment using a self-spiraling brake based on the pre-processing experimental simulation. This study will help researchers better understand the effects of 4D-printed microstructures and shed light on the multifunctional structural design and simulation of such objects.

## Data Availability

The datasets used and/or analysed during the current study available from the corresponding author on reasonable request.

## References

[1] Lendlein A, Behl M, Hiebl B, Wischke C (2010). Shape-memory polymers as a technology platform for biomedical applications. Expert Rev. Med. Devices.

[2] Tibbits S, McKnelly C, Olguin C, Dikovsky D, Hirsch S (2014). 4D printing and universal transformation. Mater. Sci..

[3] Ge Q, Sakhaei AH, Lee H, Dunn CK, Fang NX, Dunn ML (2016). Multimaterial 4D printing with tailorable shape memory polymers. Sci. Rep..

[4] Cheng T, Thielen M, Poppinga S (2021). Bio-inspired motion mechanisms: Computational design and material programming of self-adjusting 4D-printed wearable systems. Adv. Sci..

[5] Wang YN, Li X (2021). 4D printing reversible actuator with strain self-sensing function via structural design. Compos. Part B-Eng..

[6] Krüger F, Thierer R, Tahouni Y (2021). Development of a material design space for 4D-printed bio-inspired hygroscopically actuated bilayer structures with unequal effective layer widths. Biomimetics.

[7] Burgert I, Fratzl P (2009). Actuation systems in plants as prototypes for bioinspired devices. Biomimetics.

[8] Gladman AS, Matsumoto EA, Nuzzo RG, Mahadevan L, Lewis JA (2016). Biomimetic 4D printing. Nat. Mater..

[9] Ge Q, Westbrook KK, Mather PT, Dunn ML, Qi HJ (2013). Thermomechanical behavior of a two-way shape memory composite actuator. Smart Mater. Struct..

[10] Erb RM, Sander JS, Grisch R, Studart AR (2013). Self-shaping composites with programmable bioinspired microstructures. Nat. Commun..

[11] Mehrpouyaa M, Vahabib H, Janbaz S (2021). 4D printing of shape memory polylactic acid (PLA). Polymer.

[12] Zhang Q, Zhang K, Gengkai Hu (2016). Smart three-dimensional lightweight structure triggered from a thin composite sheet via 3D printing technique. Sci. Rep..

[13] Yuan C, Wang T, Dunn ML, Qi HJ (2017). 3D printed active origami with complicated folding patterns. Int. J. Precis. Eng. Manuf.-Gr. Technol..

[14] Rastogi P, Kandasubramanian B (2019). Breakthrough in the printing tactics for stimuli-responsive materials: 4D printing. Chem. Eng. J..

[15] van Manen T, Janbaz S, Zadpoor AA (2017). Programming 2D/3D shape-shifting with hobbyist 3D printers. Mater. Horiz..

[16] Felton SM, Becker KP, Aukes DM, Wood RJ (2015). Self-folding with shape memory composites at the millimeter scale. J. Micromech. Microeng..

[17] Wang YN, Li X (2021). 4D-printed bi-material composite laminate for manufacturing reversible shape-change structures. Compos. Part B-Eng..

[18] Deng H, Zhang C, Su JW, Xie Y, Zhang C, Lin J (2018). Bioinspired multi-responsive soft actuators controlled by laser tailored graphene structures. J. Mater. Chem. B.

[19] Tao R, Ji LT, Fang DN (2020). 4D printed origami metamaterials with tunable compression twist behavior and stress-strain curves. Compos. Part B-Eng..

[20] Wang D, Wang L, Wu J, Ye H (2019). An experimental study on the dynamics calibration of a 3-DOF parallel tool head. IEEE/ASME Trans. Mechatron..

[21] Ge Q, Dunn CK, Qi HJ, Dunn ML (2014). Active origami by 4D printing. Smart Mater. Struct..

[22] Ryan KR, Down MP, Banks CE (2021). Future of additive manufacturing: Overview of 4D and 3D printed smart and advanced materials and their applications. Chem. Eng. J..

[23] Ge Q, Qi HJ, Dunn ML (2013). Active materials by four-dimension printing. Appl. Phys. Lett..

[24] Wang W, Yu CY, Ahnetl SH (2019). Soft grasping mechanisms composed of shape memory polymer based self-bending units. Compos. Part B-Eng..

[25] Jeong KU, Jang JH, Kim DY, Nah C, Lee JH, Lee MH, Thomas EL (2011). Three-dimensional actuators transformed from the programmed two-dimensional structures via bending, twisting and folding mechanisms. J. Mater. Chem..

[26] Peraza-Hernandez E, Hartl D, Galvan E, Malak R (2013). Design and optimization of a shape memory alloy-based self-folding sheet. J. Mech. Des..

[27] Zeng S, Feng Y, Gao Y, Zheng H, Tan J (2021). Layout design and application of 4D-printing bio-inspired structures with programmable actuators. Bio-Des. Manuf..

[28] Murphy SV, De Coppi P, Atala A (2020). Opportunities and challenges of translational 3D bioprinting. Nat. Biomed. Eng..

[29] Qiu H, Feng Y, Gao Y, Zeng S, Tan J (2021). The origami inspired design of polyhedral cells of truss core panel. Thin-Walled Struct..

[30] Westbrook KK, Kao PH, Castro F, Ding Y, Qi HJ (2011). A 3D finite deformation constitutive model for amorphous shape memory polymers: A multi-branch modeling approach for nonequilibrium relaxation processes. Mech. Mater..

[31] Monzón MD, Paz R, Pei E, Ortega F, Suárez LA, Ortega Z, Clow N (2017). 4D printing: Processability and measurement of recovery force in shape memory polymers. Int. J. Adv. Manuf. Technol..

[32] Zhang KL, Cheng XD, Zhang YJ, Chen M, Chen H, Yang Y, Fang D (2018). Weather-manipulated smart broadband electromagnetic metamaterials. ACS Appl. Mater. Interfaces.

[33] Belhabib S, Guessasma S (2017). Compression performance of hollow structures: From topology optimisation to design 3D printing. Int. J. Mech. Sci..

[34] Feng Y, Xu J, Zeng S, Gao Y, Tan J (2020). Controlled self-spiraling deformation of programmable bilayer structures: Design and fabrication. Smart Mater. Struct..

[35] Zeng S, Gao Y, Feng Y, Zheng H, Qiu H, Tan J (2019). Programming the deformation of a temperature-driven bilayer structure in 4D printing. Smart Mater. Struct..

[36] Hawkes E, An B, Benbernou NM, Tanaka H, Kim S, Demaine ED, Wood RJ (2010). Programmable matter by folding. Proc. Natl. Acad. Sci..

[37] van Manen T, Janbaz S, Zadpoor AA (2018). Programming the shape-shifting of flat soft matter. Mater. Today.

[38] Shiblee MNI, Ahmed K, Kawakami M, Furukawa H (2019). 4D printing of shape-memory hydrogels for soft-robotic functions. Adv. Mater. Technol..

[39] Goo B, Hong CH, Park K (2020). 4D printing using anisotropic thermal deformation of 3D-printed thermoplastic parts. Mater. Des..

[40] Ding Z, Weeger O, Qi HJ, Dunn ML (2018). 4D rods: 3D structures via programmable 1D composite rods. Mater. Des..

[41] https://github.com/Ultimaker/CuraEngine. Accessed 16 May 2021.

[42] https://ultimaker.com/software/ultimaker-cura. Accessed 16 May 2021.

[43] Timoshenko S (1925). Analysis of bi-metal thermostats. Josa.

[44] Jin Y, Gao Q, Xie C, Li G, Du J, Fu J, He Y (2020). Fabrication of heterogeneous scaffolds using melt electrospinning writing: Design and optimization. Mater. Des..

[45] Jiang Y, Leng J, Zhang J (2021). A high-efficiency way to improve the shape memory property of 4D-printed polyurethane/polylactide composite by forming in situ microfibers during extrusion-based additive manufacturing. Addit. Manuf..

